# Synergy of topoisomerase and structural-maintenance-of-chromosomes proteins creates a universal pathway to simplify genome topology

**DOI:** 10.1073/pnas.1815394116

**Published:** 2019-04-08

**Authors:** Enzo Orlandini, Davide Marenduzzo, Davide Michieletto

**Affiliations:** ^a^Dipartimento di Fisica e Astronomia “Galileo Galilei,” Sezione Istituto Nazionale di Fisica Nucleare, Università degli Studi di Padova, I-35131 Padova, Italy;; ^b^School of Physics and Astronomy, University of Edinburgh, Edinburgh EH9 3FD, United Kingdom

**Keywords:** genome topology, SMC proteins, topoisomerase, Brownian dynamics, entanglements

## Abstract

Topological entanglements severely interfere with important biological processes. For this reason, genomes must be kept unknotted and unlinked during most of a cell cycle. Type II topoisomerase (TopoII) enzymes play an important role in this process but the precise mechanisms yielding systematic disentanglement of DNA in vivo are not clear. Here we report computational evidence that structural-maintenance-of-chromosomes (SMC) proteins—such as cohesins and condensins—can cooperate with TopoII to establish a synergistic mechanism to resolve topological entanglements. SMC-driven loop extrusion (or diffusion) induces the spatial localization of essential crossings, in turn catalyzing the simplification of knots and links by TopoII enzymes even in crowded and confined conditions. The mechanism we uncover is universal in that it does not qualitatively depend on the specific substrate, whether DNA or chromatin, or on SMC processivity; we thus argue that this synergy may be at work across organisms and throughout the cell cycle.

## Significance

Vital biological processes such as gene transcription and cell division may be severely impaired by inevitable entanglements ensuing from the extreme length and confinement of the genome. The family of topoisomerase proteins has independently evolved in different organisms to resolve these topological problems, yet no existing model can explain how topoisomerase alone can reduce the topological complexity of DNA in vivo. We propose that a synergistic mechanism between topoisomerase and a family of slip-link–like proteins called structural maintenance of chromosomes (SMC) can provide a pathway to systematically resolve topological entanglements even under physiological crowding and confinement. Given the ubiquity of topoisomerase and SMC, we argue that the uncovered mechanism is at work throughout the cell cycle and across different organisms.

Genomes are long polymers stored in extremely crowded and confined environments; the ensuing inevitable entanglements are thought to cause DNA damage, interfere with gene transcription and DNA replication, and interrupt anaphase, eventually leading to cell death (1–3). In vitro and under dilute conditions, type II topoisomerase (TopoII) proteins efficiently resolve topological entanglements and stabilize a population of knotted DNA below the expected value in thermodynamic equilibrium (4). These findings can be partially explained by a model where TopoII enzymes recognize specific DNA–DNA juxtapositions (5–7). However, how this model can lead to efficient unknotting and unlinking in crowded environments and crumpled DNA or chromatin substrates is unclear (2, 8, 9). Even more intriguing is the in vitro experimental finding that, in the presence of polycations (10) or with superstochiometric abundance of TopoII (11), the action of these proteins may increase the topological complexity of DNA substrates (10, 12, 13).

While it has been suggested that DNA supercoiling may provide a solution for this problem by promoting hooked DNA juxtapositions (14–16), this argument is valid only for naked, highly supercoiled DNA, such as bacterial plasmids. The understanding of how efficient topological simplification is achieved in eukaryotes where the genome is packaged into chromatin remains, on the other hand, an outstanding and unresolved problem (1, 17).

Here we propose a mechanism for efficient topological simplification in DNA and chromatin in vivo that is based on the synergistic action of structural-maintenance-of-chromosomes (SMC)-driven loop extrusion (18–21) [or diffusion (22)] and TopoII. We show that the sliding of slip-link–like proteins along DNA and chromatin is sufficient to localize any knotted and linked regions or their essential crossings, in turn catalyzing their topological simplification. Our simulations reveal that this mechanism is independent of either substrate condensation or crowding and is therefore likely to lead to unknotting and unlinking even under extreme conditions such as those in the cell nucleus. Finally, we discuss our model in the context of recent experiments reporting that SMC proteins are essential to achieve correct sister chromatid decatenation in metaphase (23), that DNA damage is frequently found in front of cohesin motion (24), and that there is a remarkable low frequency of knots in intracellular chromatin (17).

## Results and Discussion

### Model and System Setup.

**Fig. 1.**
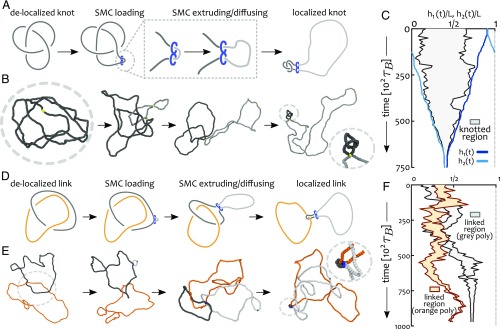
Sliding of SMC proteins localizes topological entanglements. (*A*) Schematics of knot localization starting from a fully delocalized trefoil via loop extrusion/diffusion. (*B*) Corresponding Brownian dynamics simulations. (*C*) Kymograph showing the shortest knotted arc along the chain as a function of time. The blue curves show the position of the SMC heads ($h_{1}\left(t\right),h_{2}\left(t\right)$) and demonstrate that the knot localizes over time. (*D*) Schematics of link localization starting from a delocalized Hopf link. (*E*) Corresponding Brownian dynamics simulations. (*F*) Kymograph showing the shortest linked segments for the two polymers. As the SMC protein is loaded on the gray polymer, the linked region in the sister strand is free to slide and this gives rise to a localized but fluctuating orange-shaded area (Movies S1 and S2).

We perform Brownian dynamics (BD) simulations of a generic polymer substrate modeled as a semiflexible bead-spring circular chain of 500 beads of size σ, taken to be 2.5 nm for DNA (25) and 10 nm for chromatin (26). We consider circular chains as representative of DNA plasmids or stably looped genomic regions such as the so-called “topologically associated domains” (TADs) bound by CTCF proteins (27) and knotted and linked topologies as capturing topological entanglements that typically occur in genetic materials (8, 17, 28–30) (Fig. 1). Unlike in previous works (31, 32), here we explicitly forbid spontaneous strand-crossing events by imposing that any pair of consecutive beads are connected by finitely extensible (FENE) springs (33) while nonconsecutive ones are subject to a purely repulsive (Weeks–Chandler–Andersen) potential. A Kratky–Porod term is used to set up the persistence length at $l_{p}=20\sigma$. Note, however, that the results are not qualitatively affected by this choice (*SI Appendix*).

### A Slip-Link Model for SMC.

SMC proteins, including condensin and cohesin, are thought to regulate genome architecture across organisms by topologically embracing DNA or chromatin in a slip-link–like fashion (18, 21, 34–36). Recent experiments in vitro suggest that condensin can move directionally at a speed v≃0.6−1.5 kb/s (37) and that cohesin performs diffusive sliding with diffusion constant $D≃0.1-1\;\mu m^{2}/s$ (38, 39). Previous work has crudely modeled SMC proteins as harmonic springs between nonconsecutive chromosome segments which were dynamically updated (irrespective of local constraints) to extrude loops (20, 32, 40). In contrast, here we account for both the steric hindrance and the slip-link nature of the SMC complex by modeling the SMC bond with a FENE spring so that it is energetically very unfavorable for a third segment to cross through the gap in between the bonded beads. The two chromosome segments bound by the SMC protein at time t, or SMC “heads,” are denoted as $h_{1}\left(t\right)$ and $h_{2}\left(t\right)$ and updated at rate κ (*SI Appendix*). We here focus on processive complexes and thus update the location of the heads as $h_{1}\left(t+dt\right)=h_{1}\left(t\right)+1$ and $h_{2}\left(t+dt\right)=h_{2}\left(t\right)-1$ only if the Euclidean distance between the next pair of beads is shorter than 1.3σ. This rule ensures that no third bead can pass through the segments bonded by the SMC protein during the update step and it effectively slows down the processivity of the complex, depending on the instantaneous substrate conformation. We highlight that the speed of the extrusion process does not qualitatively affect the synergistic mechanism found here, only its overall completion time.

### SMC Sliding Localizes Topological Entanglements.

Thermally equilibrated knotted or linked polymers in good solvent display weakly localized topological entanglements (41, 42); i.e., the shortest arc that can be defined as knotted or linked, $l_{K}$, grows sublinearly with the overall contour length L, as $l_{K}∼L^{0.75}$ (Fig. 1*A*) (43, 44). Further topological delocalization is achieved by isotropic confinement (45) and crowding (46), both conditions that are typically found in vivo. Since delocalization of essential crossings is likely to hinder TopoII-mediated topological simplification, it is natural to ask whether there exists a physiological mechanism that counteracts topological delocalization in vivo.

To address this question we performed BD simulations of directed loop extrusion on thermalized polymers which display delocalized entanglements (Fig. 1*A*). The ensuing extrusion, or growth, of the subtended loop can be monitored by tracking the location of the SMC heads $h_{1}\left(t\right)$ and $h_{2}\left(t\right)$ (blue curves in Fig. 1*C*). At the same time, we used well-established existing algorithms (44, 45) [publicly accessible through the server kymoknot.sissa.it (47)] to compute the shortest portion of the chain hosting the knot. We observed that the shortest knotted arc $l_{K}$, initially spanning a large portion of the polymer, progressively shrinks into a region whose boundaries match the location of the SMC heads. Notably, in the large time limit, all of the essential crossings forming the knot (in Fig. 1 a trefoil, $3_{1}$) were observed to be localized within a segment l≪L (Fig. 1*B*). A similar localization effect could be achieved on a pair of linked polymers (Fig. 1 *D*–*F*).

Importantly, SMC-driven topological localization does not require a topologically closed (circular) substrate to function. Physiologically occurring loops, e.g., between enhancer and promoters (48), CTCFs at TAD boundaries (27), or protein bridges (49), define transient and stably looped genomic regions which would effectively act as circular substrates and entrap topological entanglements such as knots and links.

### A Model for SMC-Recruited TopoII.

**Fig. 2.**
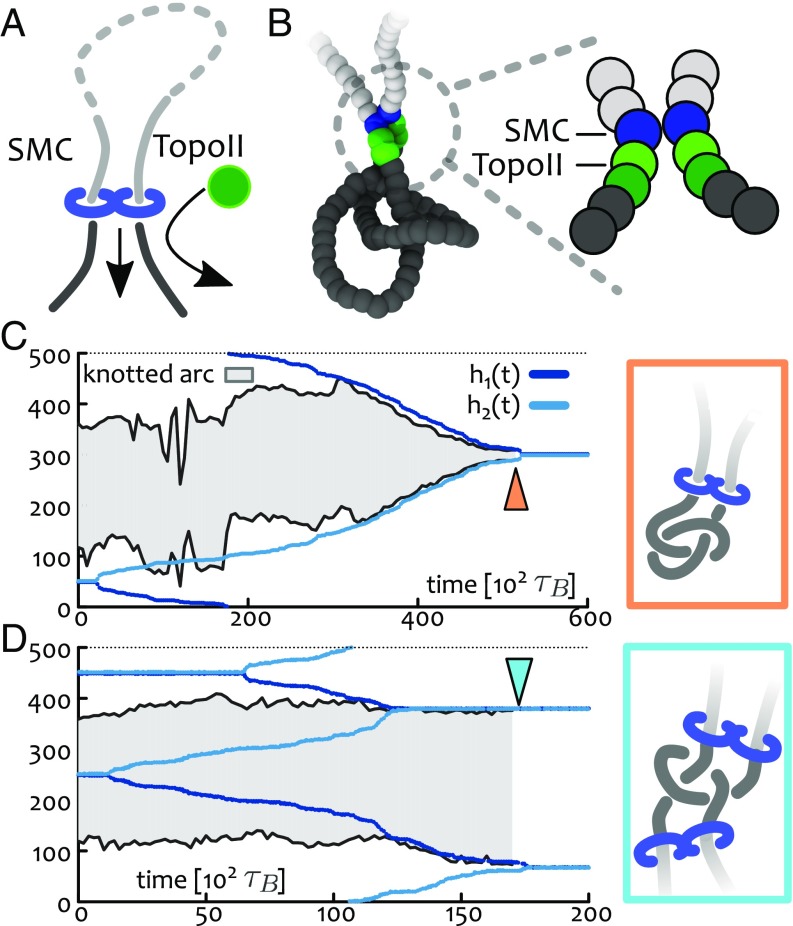
SMC-recruited TopoII. (*A*) Motivated by experimental findings (24, 50), we assume that TopoII is colocalized with SMC and it is found on the outside of the SMC-mediated loop (dark-colored segments). (*B*) Implementation of *A* in a bead-spring polymer model: The SMC slip link is enforced by a FENE bond between blue beads which are updated in time. TopoII beads (green) are set to display a soft repulsive potential with all other beads thus allowing thermally activated strand crossing. Dark green and light green beads have different energy barriers against overlapping ($5k_{B}T$ and $20k_{B}T$, respectively). (*C* and *D*) Kymographs showing synergistic knot simplification. In *C*, SMC-driven loop extrusion localizes the shortest knotted arc while in *D*, two SMCs localize only the knot’s essential crossings (*Insets*). We find that *D* is predominant for diffusive SMC (*SI Appendix* and Movies S3 and S7).

Having shown that SMC complexes can induce the localization of topological entanglements, we next asked whether downstream action of TopoII on localized entanglements could provide a fast and efficient mechanism for topological simplification. To this end, here we propose a model in which TopoII is directly recruited by SMC (Fig. 2*A*) and is motivated by recent experiments reporting direct interaction between TopoII and SMC cohesin in vivo (24, 50). Our model is qualitatively different from random passage models for TopoII (32, 51, 52) and it is practically implemented by allowing only the two nearest beads in front of the ones forming the SMC heads, i.e., $h_{1/2}\left(t\right)\pm 1$, $h_{1/2}\left(t\right)\pm 2$, to undergo strand crossing (*SI Appendix* and Fig. 2*B*).

### Localizing Topological Entanglements Catalyzes TopoII-Mediated Simplification.

We first tested whether the local recruitment of TopoII by SMC can efficiently simplify the substrate topology. To this end, we performed BD simulations initialized from equilibrated configurations containing a delocalized trefoil knot ($3_{1}$) and loaded one SMC protein recruiting a TopoII enzyme, as discussed in the previous section (Fig. 2). We monitored the time evolution of the substrate topology by computing its instantaneous Alexander polynomial (47) while tracking both the position of the SMC heads and the boundaries of the knotted region (44). Remarkably, in all of the independent replicas of the system, the synergy of SMC and TopoII was able to simplify the topology of the substrate down to the unknot (Fig. 2). Importantly, the topological simplification occurred only after the knot localization by the single SMC protein (Fig. 2*C*). To explain this finding one may argue that a localized knot enhances intraknot contacts over ones occurring between any other two segments of the polymer; in turn, this conformational bias favors the crossing of intraknot segments and catalyzes the decrease in topological complexity. Equivalently, one may recall that the probability of finding an unknot in equilibrium is exponentially small with the substrate length L, i.e., $P_{0}∼e^{-L/L_{0}}$ (53); inducing knot localization effectively yields $L<L_{0}$, thus greatly enlarging the statistical weight of unknotted conformations.

By loading more than one SMC protein onto the substrate we discovered that there exists another pathway for topological simplification. This involves the localization of the essential crossings but does not lead to a minimal knotted arc $l_{K}\ll L$; this pathway is selected when a pair of SMCs extrude loops simultaneously from within and outside the knotted region (Fig. 2*D*) and it yields polymer conformations that are reminiscent of those computationally observed in DNA knot translocation (54). Interestingly, this unknotting pathway is favored and often observed in simulations of diffusing slip links (*SI Appendix* and Movie S7).

For simplicity, we assumed an infinitely long residency time of SMC proteins. While a population of condensins is stably bound in mitosis (55), cohesin is known to turn over in about τ=20 min through interphase (56). At a speed v≃1 kb/s (37), SMC proteins can extrude loops of length l=vτ>Mb during their lifetime. By diffusing at $D≃0.1-1\;\mu m^{2}/s$ (38, 39) SMC proteins can cover distances of about $\sqrt{D\tau }≃200-700\;kb$ over a loosely packed chromatin storing 200 bp in 10 nm (*SI Appendix*). In either case the processivity [p=vτ (20) or $p=\sqrt{D\tau }$ (22)] of the SMC is comparable to (or larger than) both the length of typical TADs—which have median 185 kb in humans (57)—and that of our polymer substrate (200–500 kb). In *SI Appendix*, we show that when the SMC processivity is shorter than the length of the substrate, our synergistic model can still achieve topological simplification, albeit in a stochastic sense.

We finally highlight that the observed topological simplification is different from all existing alternative mechanisms accounting for the action of TopoII alone (51, 58). Our mechanism also works in the absence of high levels of supercoiling, known to provide another nonequilibrium pathway for postreplicative decatenation (16), but not documented in eukaryotic chromatin.

### Synergistic Topological Simplification Is Efficient in Crowded and Confined Conditions.

**Fig. 3.**
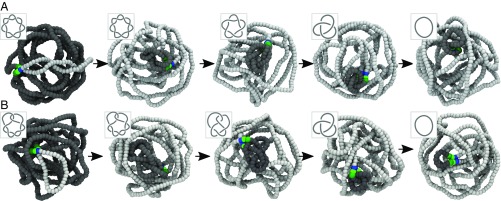
Efficient unknotting under confinement. The synergistic action of SMC and TopoII proteins can systematically simplify knotted substrates even under confinement. Here we show the case of torus ($7_{1}$) and twist ($7_{2}$) knots confined within a sphere with radius $R_{c}/\left\langle R_{g}\right\rangle ≃1/3$. In the snapshots, light gray beads are the ones that have been extruded by, hence behind, the SMC. Dark gray beads are the ones outside the extruded loop. Blue beads mark the location of the SMC heads. Green and dark green beads mark the location of TopoII, as described in the text. (*A*) Unknotting of a $7_{1}$ knot through the “cascade” of torus knots $5_{1}$ and $3_{1}$. (*B*) Unknotting of a $7_{2}$ knot through $5_{2}$ and $3_{1}$ knots. Direct simplification $7_{2}\;\to \;0_{1}$ is also observed in more than half of the simulations (Table 1, *SI Appendix*, and Movies S4 and S5).

One of the major problems in elucidating TopoII-mediated topological simplification in vivo is that it must “recognize” the global topology of the substrate while performing local strand crossings. Hooked DNA juxtapositions between prebent segments may provide a simple readout mechanism to simplify localized knots in dilute conditions (5, 51, 59). However, this is not a viable pathway in crowded or confined conditions such as those in vivo because (*i*) in dense solutions many DNA–DNA juxtapositions occur by random collision regardless of the local bending and (*ii*) knots and other forms of topological entanglement tend to delocalize under isotropic confinement (45). It is thus natural to ask whether the synergistic mechanism proposed here may provide a robust pathway to simplify genome topologies under confinement, as required within the nucleus of cells. To this end we performed simulations on polymers displaying a range of knot types and confined within a sphere of radius $R_{c}$ about three times smaller than the mean gyration radius of the same polymer in equilibrium in good solvent, $\left\langle R_{g}\right\rangle$. Remarkably, we discovered that the synergistic action of SMC and TopoII can efficiently simplify the substrate topology even in this extreme confinement regime. In particular, as the SMC protein slides along the crumpled substrate, we observed configurations in which a third segment is found in front of the extruding fork (Fig. 3), highly reminiscent of hooked juxtapositions (58, 59). Within our model, these events are spontaneous, in that they are due to the linear reeling in of the substrate through the SMC slip link. These findings also suggest that the recruitment of TopoII in front of the extruding motion of the SMC (50) may be an evolutionarily optimal strategy to resolve topological entanglements.

Differently from other models, the mechanism we propose here can achieve efficient topological simplification under confinement and our simulations even suggest that our model may be the more efficient the stronger the confinement (*SI Appendix*). This can be explained as the entropic penalty for forming a loop of size l by the SMC complex scales as $ck_{B}T\log l$ with c the contact exponent (22, 60, 61). Thus, on crumpled substrates, i.e., c≃1, the entropic penalty is smaller than on swollen ones, c≃2.1. This implies that the extrusion/diffusion of the SMC is less hindered under confinement and the localization of the knot is thus achieved more quickly (*SI Appendix*, Fig. S4).

### Comparison of the Synergistic vs. Random Passage and Hooked Juxtaposition Models.

To compare the efficiency of the mechanism proposed here against that of previous models for TopoII, we estimated the transition probabilities within the space of knots, $P\left(K_{1}\to K_{2}\right)$ by performing 50 simulations starting from equilibrated polymers tied in a range of different knots. Some of the transition probabilities are reported in Table 1, for both free and confined polymers, and are compared with those reported by random passage (RP) (51, 62) and hooked juxtaposition (HJ) (59) models (complete table in *SI Appendix*). The transition rates toward simpler topologies outperform those of other TopoII-only models, in particular for more complex knots. For instance, to unknot a $7_{1}$ we predict the cascade $7_{1}\;\to \;5_{1}\;\to \;3_{1}\;\to \;0_{1}$ with probability $P\left(7_{1}\;\to \;0_{1}\right)=P\left(7_{1}\;\to \;5_{1}\right)P\left(5_{1}\;\to \;0_{1}\right)P\left(3_{1}\;\to \;0_{1}\right)=0.98$, which is about 12 times larger than the one predicted by RP models (0.082). This enhanced simplification with respect to RP and HJ models increases with knot complexity and with the degree of confinement. For instance, under the confinement chosen here, the RP model would predict a probability $P\left(7_{1}\;\to \;5_{1}\;\to \;3_{1}\;\to \;0_{1}\right)≃0.002$ that is about 300 times smaller than the one achieved by our synergistic model (0.75).

**Table 1.** Knot transition probabilities in different models

	Synergistic		RP		HJ
Knot transitions	Free, transitions	Confined, this work	Free (62)	Confined, this work	Free (59)
$7_{1}\;\to \;K$	0.02	0.06	0.66	0.98	—
$7_{1}\;\to \;5_{1}$	0.98	0.92	0.34	0.02	—
$7_{1}\;\to \;3_{1}$	0	0.02	0	0	—
$5_{2}\;\to \;K$	0	0.1	0.49	0.8	0.26
$5_{2}\;\to \;3_{1}$	0.5	0.25	0.2	0.13	0.23
$5_{2}\;\to \;0_{1}$	0.5	0.65	0.31	0.07	0.51
$5_{1}\;\to \;K$	0	0.06	0.69	0.8	—
$5_{1}\;\to \;3_{1}$	1	0.94	0.31	0.13	—
$4_{1}\;\to \;K$	0	0.04	0.16	0.84	—
$4_{1}\;\to \;0_{1}$	1	0.96	0.84	0.16	—
$3_{1}\;\to \;K$	0	0.15	0.22	0.87	0.2
$3_{1}\;\to \;0_{1}$	1	0.85	0.78	0.13	0.8

Topology simplification through the synergistic model proposed in this work is compared with that in RP (62) and HJ (59) models. The confined case is compared with RP simulations performed in this work. $K_{1}\to K$ denotes transition to any knot K with equal or larger minimal crossing number. Complete table is given in *SI Appendix*.

### Randomly Bound vs. SMC-Localized TopoII.

While recent experimental data on SMC cohesin support our hypothesis of TopoII–SMC colocalization (24, 50), such evidence is poorer for condensin and bacterial SMC. Thus, we tested whether a model in which TopoII is dynamically and randomly associated with the polymer during SMC extrusion can still yield efficient topological simplification. We performed simulations of a confined trefoil in which a random fraction of contour length ϕ is allowed to undergo strand-crossing events and set the turnover time for TopoII-bound segments to be comparable to that taken to extrude one persistence length (*SI Appendix*).

**Fig. 4.**
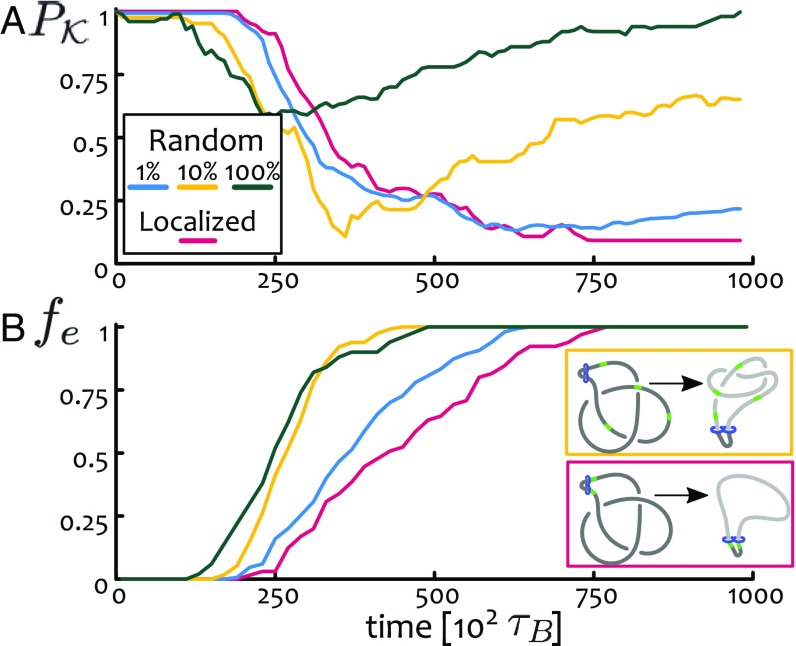
Localized vs. random TopoII under confinement. We perform simulations on a trefoil under confinement $R/\left\langle R_{g}\right\rangle =1/3$ and measure (*A*) the knotting probability $P_{K}$ and (*B*) the fraction of completed loops $f_{e}$ as a function of time. Our results show that while models of randomly bound TopoII can lead to substrate unknotting, they entail a return to equilibrium values of $P_{K}$ once SMCs stop extruding.

We discovered that the knotting probability $P_{K}$ shows a nonmonotonic behavior as a function of time for all models of randomly associated TopoII (Fig. 4*A*). By measuring the fraction of fully extruded loops $f_{e}$ we observed that the recovery of $P_{K}$ at large times occurs after $f_{e}≃1$. This is to be expected, since models with randomly associated TopoII must return to the equilibrium value for pure random passage events with ϕ-dependent kinetics. On the contrary, in our original model where TopoII is localized only at the SMC, the successfully extruded polymer segments are no longer able to cross each other and the topology is thus fixed at all future times. Thus, the recovery of $P_{K}$ to its equilibrium values is neither expected nor observed. We thus argue that for randomly bound thermally activated TopoII a continuous flux of dynamically associated SMC is required to maintain a knotting probability below equilibrium.

## Conclusions

In this work, we have provided numerical evidence for a molecular mechanism that can efficiently maintain genomes free of entanglements. This is based on the combined action of SMC-driven extrusion and TopoII-mediated strand crossing. The sliding of molecular slip links along knotted or linked substrates naturally generates highly localized entanglements (Fig. 1), in turn catalyzing their simplification through TopoII (Fig. 2), also under strong confinement (Fig. 3). Importantly, the envisaged mechanism is universal, in that it works equally well on DNA or chromatin, closed plasmids or stably looped linear genomic regions such as TADs, interphase and mitosis, and across all life forms that have evolved TopoII-like and SMC-like proteins.

Our findings show that SMC proteins are indispensable to correctly decatenate sister chromatids, in agreement with experiments (63–65), and also shed light on recent findings reporting the accumulation of DNA damage in front of cohesin complexes (24). We argue that the sliding motion of SMC entraps topological entanglements, in turn increasing local stresses that may lead to double-strand DNA breaks. Our results thus provide compelling mechanistic evidence for an evolutionarily optimal strategy whereby TopoII is actively recruited by SMC complexes (50). At the same time, we showed that randomly bound TopoIIs can still yield efficient topological simplification, if combined with dynamically associated SMCs (Fig. 4).

While we here assumed unidirectional SMC motion, we expect that similar physics should be at work for diffusing SMCs (22) as the entropic competition between slip links and knots may favor the former under some conditions (66) (*SI Appendix*).

We also argue that an analogous mechanism may take place during DNA replication, whereby the polymerizing machinery effectively functions as a slip link and localizes entanglements. TopoII is known to act in front of the replication fork (67), and thus the very same synergistic mechanism for topological simplification proposed here may be at play in this context as well. It is also of interest to note that PCNA, the molecular clamp associated with a processive polymerase (68), recruits components of repair complexes, which would again be evolutionarily advantageous to resolve entanglement-related DNA damage. All of this reinforces the idea that the mechanism we propose may be universal.

We finally speculate that the remarkable low knotting probability recently quantified in intracellular chromatin and its weak or absent scaling with the length of the substrate (17) may be explained by our model as we find it to be remarkably insensitive to substrate length (*SI Appendix*). We hope that our work will ignite new experimental efforts to identify and further characterize novel synergistic mechanisms that may regulate genome topology.

We conclude this work by speculating on an open question: If TopoII can cooperate with ATP-consuming SMCs to simplify genome topology, why does it require ATP to function [as shown in vitro (4)]? A possible explanation is that the synergy between passive TopoII and active SMC would still be insufficient to maintain a functionally viable genome in the cell nucleus. We hope that either future models accounting for nonequilibrium TopoII or experiments exploring the synergy of TopoII and SMC may shed light on this intriguing problem.

## Materials and Methods

### Chromatin/DNA Model.

We use a well-established bead-spring polymer model (33) to describe chromatin and DNA (31). We account for excluded volume and chain uncrossability by using shifted and truncated Lennard-Jones interactions and finitely extensible springs to prevent thermally activated strand-crossing events as discussed in the main text (also *SI Appendix*). A publicly available code (47) is used to detect the shortest physically knotted arc within the substrate.

### Integration Procedure.

Each bead in our simulation is evolved through the Langevin equation $m_{a}\partial_{tt}{\vec{r}}_{a}=-\nabla U_{a}-\gamma_{a}\partial_{t}{\vec{r}}_{a}+\sqrt{2k_{B}T\gamma_{a}}{\vec{\eta }}_{a}\left(t\right)$, where $m_{a}$ and $\gamma_{a}$ are the mass and the friction coefficient of bead a, and ${\vec{\eta }}_{a}$ is its stochastic noise vector satisfying the fluctuation–dissipation theorem. U is the sum of the energy fields (*SI Appendix*). The simulations are performed in LAMMPS (69) with $m=\gamma =k_{B}=T=1$ and using a standard velocity Verlet algorithm.

### Note Added in Proof.

After the present paper was submitted for publication, we learned of a similar model simultaneously developed by Racko et al. (70).

## Supplementary Material

Supplementary File

Supplementary File

Supplementary File

Supplementary File

Supplementary File

Supplementary File

Supplementary File

Supplementary File
