# Supplementary material for: Synergy of topoisomerase and structural-maintenance-of-chromosomes proteins creates a universal pathway to simplify genome topology
Source: Proc Natl Acad Sci U S A. 2019 Apr 8;116(17):8149–54. doi: 10.1073/pnas.1815394116 (PMC6486742; doi:10.1073/pnas.1815394116)
Supplement: Supplementary File [file pnas.1815394116.sapp.pdf]

Synergy of Topoisomerase and SMC Proteins Creates a Universal Pathway to Simplify Genome Topology: Supplementary Information

Enzo Orlandini,[1] Davide Marenduzzo,[2] and Davide Michieletto*[2]

[1] *Department of Physics and Astronomy, University of Padova, Via Marzolo 8, I-35131 Padova, Italy*
[2] *School of Physics and Astronomy, University of Edinburgh, Peter Guthrie Tait Road, Edinburgh, EH9 3FD, UK*

I. COMPUTATIONAL DETAILS

A Polymer Model for Chromatin and DNA substrates

We model a polymer substrate, such as DNA or chromatin, as a chain of beads of size σ connected by springs. This types of models are widely employed in the literature and have been shown to faithfully capture the physical behaviour of DNA and chromatin [1–4]. To ensure that the polymer substrates does not cross through itself, we impose that any two beads (a, b) at distance r are subject to a purely repulsive (WCA) potential

$$U^{ab}_{WCA}(r) = k_B T \left[4 \left[\left(\frac{\sigma}{r}\right)^{12} - \left(\frac{\sigma}{r}\right)^{6} \right] + 1 \right] \text{ if } r \leq 2^{1/6}\sigma \tag{1}$$

and 0 otherwise. Further, we impose that consecutive beads are connected by finitely extensible (FENE) springs modelled as

$$U^{ab}_{FENE}(r) = -\frac{k_f R_0^2}{2} \ln \left[1 - \left(\frac{r}{R_0}\right)^2 \right] \text{ if } r \leq R_0 \tag{2}$$

and ∞ otherwise. Here, $k_f = 30\epsilon/\sigma^2$ and $R_0 = 1.5\sigma$ are typical parameters employed to prevent spontaneous chain crossing [5]. We account for DNA or chromatin stiffness by adding a potential controlling the angle formed by consecutive triplets of beads

$$U^{ab}_{KP} = \frac{k_B T l_p}{\sigma} \left[1 - \frac{\boldsymbol{t}_a \cdot \boldsymbol{t}_b}{|\boldsymbol{t}_a||\boldsymbol{t}_b|} \right], \tag{3}$$

where $\boldsymbol{t}_a$ and $\boldsymbol{t}_b$ are the tangent vectors connecting bead a to $a+1$ and b to $b+1$ respectively; l_p is the persistent length of the chain and by setting $l_p = 20\sigma = 50$ nm we model an average DNA sequence [6] while with $k_\theta = 3\, k_B T$ we account for a more flexible polymer with $l_p = 3\sigma = 90$ nm such as a 30nm chromatin fibre [7].

A Model for Structural Maintenance of Chromosome Proteins

The SMC proteins – such as cohesins and condensins – are a well-known and widely studied family of proteins [8] that have now been identified as responsible for dynamic genomic loops in both inter- and meta-phase [9, 10]. These proteins can be crudely viewed as physical slip-links [11] that embrace one, or two, double-stranded DNA and slide along DNA/chromatin [12, 13] in turn stabilising the formation of dynamic loops [12, 14] and halting at "anchor" points embodied by converging CTCF proteins [9, 15]. In this work, we aim to mechanistically investigate the generic effect of SMC proteins on topological entanglements – such as knots and links – that may be present on DNA or chromatin in interphase and mitosis. To this end, we propose a generic model where SMC proteins loaded on the polymer are described as bonds connecting two non-consecutive beads along the chain. Importantly, and in marked contrast with recent models of loop extrusion [2, 14, 16], here we account for the physical presence of a slip-link-like molecule joining two segments of chromosomes by forcing the maximum extension of the bond with a FENE potential so that it is energetically very unfavourable for a third bead to cross through the gap in between the joined segments. Again, this is done to prevent spontaneous events that would change the local topology of the substrate and that are not physically possible in real situations. It is worth noting that this detail had not been correctly accounted for in some of the existing models of loop extrusion [2, 14]. In other words, the SMC protein is modelled by including a potential

$$U^{h_1 h_2}_{SMC}(r) = -\frac{k_f R_0^2}{2} \ln \left[1 - \left(\frac{r(h_1, h_2)}{R_0}\right)^2 \right] \text{ if } r \leq R_0 \tag{4}$$

and ∞ otherwise, and where h_1 and h_2 are the instantaneous position of the two segments of chromosome bound by the SMC protein at time t (or the SMC "heads"). At rate $\kappa = 0.01\tau_B^{-1}$ ($\tau_B \equiv \sigma^2/D$ is the Brownian time of a bead, see below), we change the position of the heads via the following protocol:

$$\begin{cases} h_1(t+dt) = h_1(t) + 1 \text{ and} \\ h_2(t+dt) = h_2(t) - 1 \text{ if } d(h_1, h_2) \leq 1.3\sigma \\ h_1(t+dt) = h_1(t) \text{ and} \\ h_2(t+dt) = h_2(t) \text{ otherwise or if } h_1 = h_2\,. \end{cases} \tag{5}$$

Thus, the SMC enlarges the loop formed by two monomers on average every 100 Brownian times only if the distance between the next pair of beads is shorter than or equal to 1.3σ in 3D space. This choice ensures that no third bead can pass through the beads bonded by the SMC protein and it effectively slows down the speed of the complex from $v_{max} = 2\sigma\kappa$ to about $v \simeq 0.1\, v_{max}$. Unless otherwise stated, we will consider $v_{max} = 0.02\,\sigma/\tau_B$. Yet, we stress that the speed of the

*davide.michieletto@ed.ac.uk

www.pnas.org/cgi/doi/10.1073/pnas.1815394116

extrusion (or diffusion) process does not affect the efficiency of the synergistic mechanism we uncover in this work.

Mapping to Real Units

Given the size of a bead σ and the energy scale k_BT (at room temperature), we can derive the typical (Brownian) time taken for a bead to diffuse its own size as $\tau_B = \sigma^2/D = 3\pi\eta\sigma^3/k_BT$. Using the viscosity of the nucleoplasm $\eta \simeq 200cP$ [17] we obtain that our simulated Brownain time corresponds to $\tau_B = 7\mu$s for DNA and $\tau_B = 12$ ms for a 30nm chromatin fibre. The initial state of our simulations is an equilibrated polymer conformation (without SMC proteins acting on it) that is obtained running 10^5 τ_B steps, i.e. of the order of seconds for DNA and tens of minutes for chromatin. Production runs in which SMC proteins are loaded on the polymer also typically cover 10^5 τ_B steps which we find is enough for complete knot localisation.

Topoisomerase Model

In contrast to previous works which crudely model the action of TopoII as a uniform non-zero probability of strand-crossing events [1, 16, 18], here we assume that TopoII is locally recruited by the SMC protein and it is loaded on the outside of the loop subtended by the complex (see below for extensions of our model that relax this assumption). Thus, here only the two beads (about 60 nm) in front of the ones forming the SMC complex are allowed to undergo strand-crossing events. In simple terms, if $h_{1,2}(t)$ are the positions of the SMC heads then $(h_1(t)-1, h_1(t)-2)$ and $(h_2(t)+1, h_2(t)+2)$ are the beads associated to TopoII. In practice, we set the interaction of these beads with all other beads as a soft repulsion

$$U_{Topo} = A\left[1+\cos\left(\frac{\pi r}{r_c}\right)\right] . \quad (6)$$

To avoid numerical instabilities which may occur due to the dynamic update of the SMC heads, we tune A so that it displays an increasing energy gradient, i.e. the furthest bead from the SMC complex is set to have $A = 5k_BT$ while the closer one $A = 20k_BT$. This ensures that when the position of the SMC is updated, it is unlikely for two beads interacting through the WCA potential to be overlapping.

Integration Procedure

The total energy field experienced by bead a is the sum of all the pairwise and triplet interactions involving all other beads, i.e.

$$U_a = \sum_{b\neq a}\left[U^{ab}_{WCA} + \left(U^{ab}_{FENE} + U^{ab}_{bend}\right)(\delta_{b,a+1}+\delta_{b,a-1})\right] + \\ + U^{ab}_{SMC}(t)\delta_{a,h_1}\delta_{b,h_2}\, , \quad (7)$$

where the Kronecker deltas $\delta_{i,j}$ indicate that bond and angle potentials are restricted to consecutive beads along the polymer and that the SMC potential is acting on the beads corresponding to the SMC heads. The time evolution of each bead in the system is thus governed by the following Langevin equation,

$$m_a\frac{d^2\vec{r}_a}{dt^2} = -\nabla U_a - \gamma_a\frac{d\vec{r}_a}{dt} + \sqrt{2k_BT\gamma_a}\vec{\eta}_a(t), \quad (8)$$

where m_a and γ_a are the mass and the friction coefficient of bead a, and $\vec{\eta}_a$ is its stochastic noise vector obeying the following statistical averages:

$$\langle\boldsymbol{\eta}(t)\rangle = 0; \quad \langle\eta_{a,\alpha}(t)\eta_{b,\beta}(t')\rangle = \delta_{ab}\delta_{\alpha\beta}\delta(t-t'), \quad (9)$$

where the Latin indices represent particle indices and the Greek indices represent Cartesian components. The last term of Eq. (8) represents the random collisions caused by the solvent particles and, for simplicity, we assume all beads have the same mass and friction coefficient (i.e. $m_a = m$ and $\gamma_a = \gamma$) and finally set $m = \gamma = k_B = T = 1$. Equation (8) is integrated using a standard velocity-Verlet algorithm, which is performed using the Large-scale Atomic/Molecular Massively Parallel Simulator (LAMMPS) [19]. For the simulation to be efficient yet numerically stable, we set the integration time step to be (unless otherwise stated) $\Delta t = 0.01\,\tau_B$, where τ_B is the Brownian time mentioned previously.

II. CIRCULAR POLYMERS MIMIC PLASMIDS OR STABLY LOOPED GENOMIC REGIONS

Our choice to consider circular polymers as substrate for the synergistic action of SMC and TopoII is motivated by the following arguments. First, circular genomes exist in nature, for instance plasmids in bacteria and mini-rings in the kinetoplast DNA [20] (our polymers would correspond to a DNA molecule $L = N\sigma = 1.25\,\mu m \simeq 4$ kbp long if taking $\sigma = 2.5$ nm). Second, although eukaryotic genomes are not topologically closed such as bacterial plasmids, they are transiently looped by bridge proteins [21]. In particular, the size of the polymers considered here would map to $L = 500$ kbp if coarse-graining a chromatin fibre with thickness $\sigma = 10$ nm $= 1$ kbp (tightly packed chromatin) or $L = 100kbp$ if considering $\sigma = 10$ nm $= 200$ bp (loosely packed chromatin). The length of so-called "Topologically Associated Domains" (TADs) in humans ranges from 40kbp to 3Mbp with a median of 185 kbp [15]; thus the polymers considered here would represent typical TADs. Increasing evidence suggest that TADs are stably looped by CTCF complexes [15, 22, 23] and therefore any non-trivial topological state (a knot or a link) assumed by TADs would be topologically trapped as long as the TAD itself is looped.

For these reasons we argue that our choice of polymer size and global topology (that of a ring) correctly capture the length-scales and topological problem faced in vivo by bacterial and eukaryotic cells.

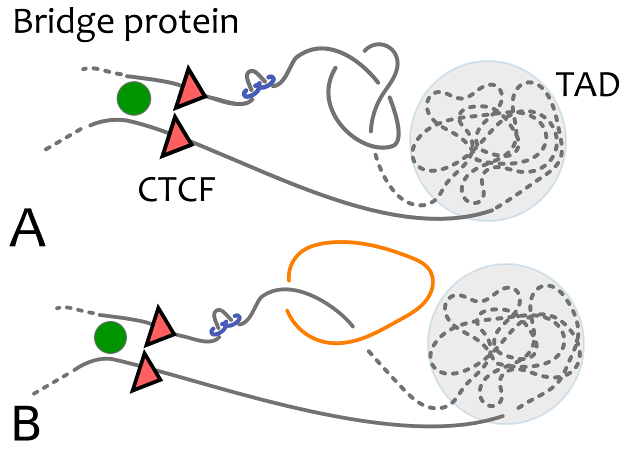

Figure S1: Considering a circular polymer as a substrate not only mimics bacterial plasmids and DNA mini-rings but also stably looped genomic regions in eukaryotes. These regions may be looped by chromatin-binding proteins such as transcription factors (e.g., HMGB2 [24]) and Polymerases, but also by CTCF [23] in the case of TADs.

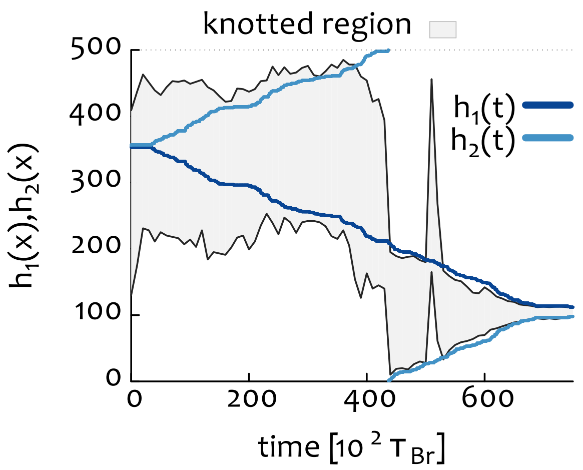

Figure S2: The figure shows a kymograph of the shortest knotted segment (grey shade and black lines) together with the instantaneous location of the SMC heads (shades of blue). In particular, this kymograph shows an event of knot inversion by loading a SMC protein within the shortest knotted arc at time 0.

III. KNOT INVERSION VIA SMC EXTRUSION

In this section we report a localisation events that starts from a SMC loading occurring within the shortest knotted arc. Such an event is not unlikely, since (i) the knot is originally delocalised and thus occupies a non-negligible contour length of the polymer and (ii) the loading of a SMC is a local event that cannot measure non-local topology. We inspected out simulations and in Fig. S2 we report a kymograph of one such an event. As one can notice, the boundaries of the knot are first inflated and then, because of globally closed topology of the underlying polymer, wrapped around and collapsed. This event thus lead to full knot localisation as the case in which the SMC is loaded outside the shortest knotted arc.

IV. LOCALISATION EFFICIENCY AS A FUNCTION OF NUMBER OF SMC

It is reasonable to ask whether the synergistic effect we uncover in this work may be made more efficient by considering multiple SMC extruding loops on the same substrate. To answer this question we perform simulations in which we simultaneously load 1, 2 and 4 SMC complexes at a random position along a polymer which is tied in a trefoil knot. The interaction between SMC heads is here considered mutually exclusive, i.e. if two SMC heads are found on consecutive beads and moving in opposite directions they remain still as cannot overlap on the same bead. In these simulations we discover two seemingly counter-intuitive effects:

1. the knots which become localised do so in shorter time when multiple SMC are loaded (Fig.S S3A);

2. the probability to find a localised knot (here practically defined as one made by less than 50 beads) at large times decreases with the number of SMC (Fig.S S3B);

The former finding can be readily explained by the fact that multiple SMC can extrude more contour length on the same unit of time. Yet, the decrease in localisation probability is more puzzling.

By close inspection of the simulation trajectories we discover that this reduction in localisation probability is due to situations in which two or more SMC proteins are simultaneously loaded within and outside a knotted region. These situations may lead to trapped conformations that stabilise a delocalised knotted state (see kymograph and snapshots Fig. S3). On the contrary, a single SMC, even if loaded within a knotted region can turn the knot "inside-out" and ultimately generate a fully localised knot.

V. UNKNOTTING IS FAVOURED UNDER CONFINEMENT

As mentioned in the main text, we find that the proposed synergistic mechanism between SMC and TopoII can simplify knots even under strong confinement. Because of this, we argue that this pathway may be at work in vivo. Here, we further characterise this finding by quantifying the rate of knot localisation as a function of confinement. To this end, we perform different sets of 40 independent simulations in which a trefoil knot tied along a $N = 300$ beads polymer is confined within a sphere of varying radius R_c and subject to the action of a single SMC. We consider a range of values for R_c ranging from tight confinement $R_c = 10\sigma \simeq \langle R_g \rangle/3$ to $R_c = 50\sigma > \langle R_g \rangle$, where $\langle R_g \rangle$ is the typical size of the polymer in equilibrium in good solvent and under no confinement.

Remarkably, we discover that the typical localisation time (here practically defined as the first time at which the shortest knotted arc spans less than 50 beads) is shorter the stronger is the confinement (see Fig. S4). We argue that this puzzling finding can be explained by the following argument: the

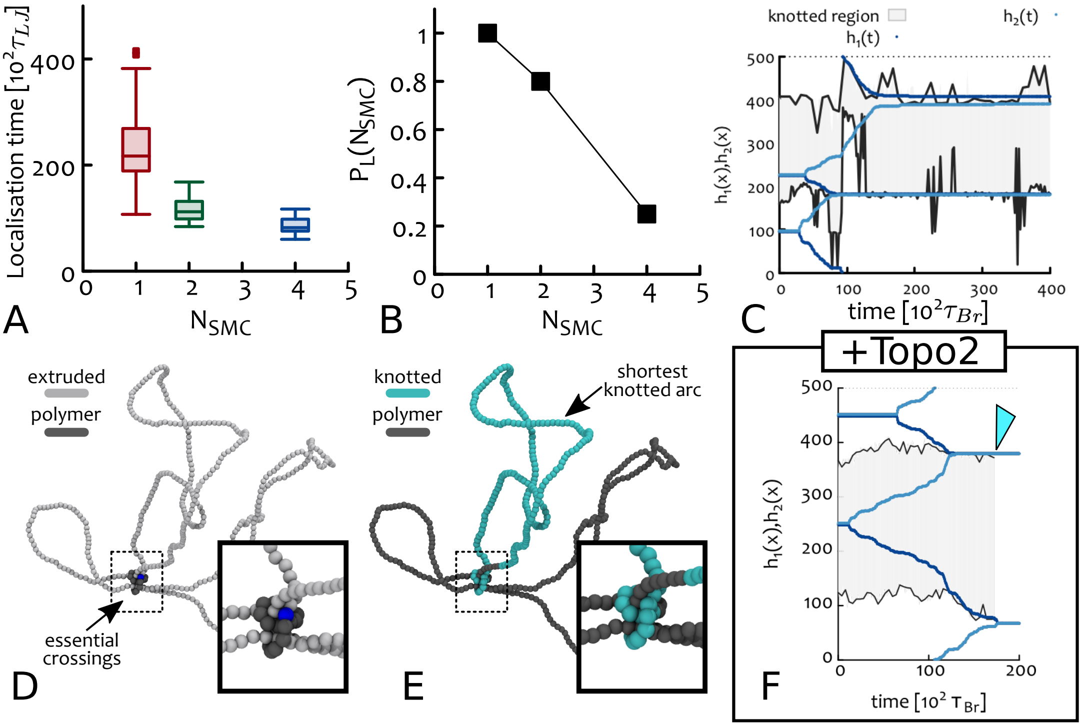

Figure S3: (A) Box plots showing the distribution of localisation times on a polymer substrate $N = 500$ beads long and with SMC moving at rate $\kappa = \tau_{Br}^{-1}$. [Here a knot is considered localised if its shortest arc spans less than 50 beads]. (B) Probability to find a localised knot at large times as a function of the number of SMC proteins simultaneously loaded on the substrate. (C) Kymograph of a simulation for a polymer tied in a trefoil knot and with 2 SMC loaded at time $t = 0$. The shortest arc that can be defined knotted (after suitable closure [25]) is shaded in grey. The 4 SMC heads are shown in shades of blue. One of the two SMC is loaded within the knotted region at time $t = 0$, whereas the other is outside it. Eventually, heads belonging to different SMC meet and stall, thereby stabilising a delocalised knot. (D) Snapshot from the same simulation used to generate (C) and showing extruded segments in light grey and the polymer backbone in dark grey. One can see that essential crossings are localised (inset). (E) Snapshot highlighting the shortest arc that can be defined knotted in blue, while the rest of the polymer is showed in dark grey. These snapshots shows that even if essential crossings are localised, the knot itself need not be. This conformation has been recently found in simulations of translocation of knotted DNA [26]). See Supplementary Movie M6. (F) Including Topo2 localised in front of SMC leads to the unknotting of a knot whose essential crossings are localised. The arrowhead in **F** indicates the unknotting event.

entropic penalty associated with the formation of a loop of length l is $S/k_B \sim c \log l$ where c is the exponent determining the decay of the contact probability $P_c(l) \sim l^{-c}$. For a crumpled polymer, i.e. the conformation assumed under confinement, the contact exponent $c = \nu d = 1$, whereas for a swollen coil in good solvent (self-avoiding), $c \simeq 2.1$ [27]. For this reason, the entropic penalty grows more steeply for a swollen coil than for a crumpled globule. In turn, this implies that the loop extruding action of the SMC protein is entropically favoured (or less hindered) under confinement, in qualitative agreement with our findings (see Fig. S4).

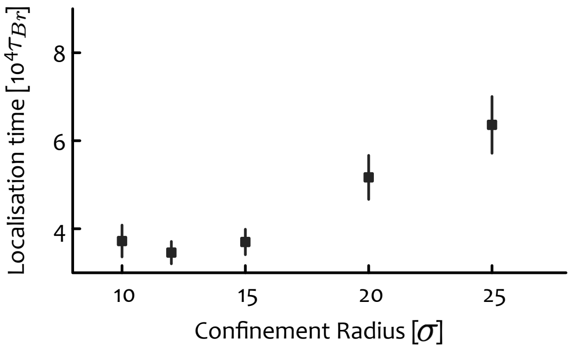

Figure S4: Average localisation time of a trefoil knot tied along a polymer $N = 300$ beads long, under confinement within a sphere and subject to the action of a single SMC moving at rate $\kappa = 0.1\tau_{Br}^{-1}$. The error bars represent the standard error of the mean.

VI. SYNERGISTIC UNKNOTTING IS INSENSITIVE TO SUBSTRATE LENGTH

In this section we provide a more quantitative, albeit not definitive, examination of efficiency of the proposed synergistic simplification as a function of the length of the substrate. Because of the largely fluctuating 3D conformations assumed by long polymers, the random passage and hooked juxtaposition models are known to be sensitive on this parameter [28]. To compare these models with the one proposed here, we perform 4 sets of 50 independent simulations starting from an equilibrated (and unconfined) polymer tied as a 7_1 knot with

	$M = 300$	$M = 500$	$M = 1000$	$M = 2000$
$7_1 \to K$	0	0.02	0	0.1
$7_1 \to 5_1$	1	0.98	1	0.9
$5_1 \to K$	0	0	0	0.03
$5_1 \to 3_1$	1	1	0.97	0.97
$5_1 \to 0_1$	0	0	0.03	0
$3_1 \to K$	0	0	0	0.03
$3_1 \to 0_1$	1	1	1	0.97

Table I: Comparison of unknotting pathways for a 7_1 knot tied along a polymer of length M in unconfined conditions and subject to the synergistic unknotting. To obtain this table, 50 independent simulations were initialised from a 7_1 state and the transition to other knot types recorded. $k_1 \to K$ denotes a transition to any knot type with minimal crossing number larger than k_1.

varying length M, ranging from 300 to 2000. If one takes $\sigma = 2.5$ nm as the diameter of DNA, then this range compares to $750 - 5000$ bp. In Table T I we report the values of the transitions $k_1 \to k_2$ observed for these different lengths of the substrate.

As one can notice, we find that for $M = 300$ the 7_1 is taken to the unknot through 5_1 and 3_1 with probability 1 and this probability is only mildly, if at all, affected for longer substrates. We highlight that this finding is likely to due to the fact that TopoII strand-crossing occurs more likely in pre-localised topological entanglements, thus strongly biasing their simplification over the increase in complexity.

In other words, while the time to localise a knot increases on longer substrates, the simplification cascade towards the unknot is virtually unaffected. In light of this insensitivity, we reason that if this mechanism is at work in vivo, then the knot probability in intracellular chromatin should only weakly depend on the length of the fibre under consideration. Intriguingly, this observation is consistent with very recent experimental findings on the knotting of chromatin fires in vivo [29].

VII. LOADING AND UNLOADING SMC COMPLEXES

SMC proteins, cohesin and condensin, have a finite residency time on chromatin [30, 31]. For cohesin, this is typically of the order of $\tau = 20$ minutes [32]. On the contrary, the model considered up to now assumed that SMC would never disassociate from the substrate. This assumption is well justified only in the regime in which SMC proteins cover a length at least equal to the polymer size L before disassociating. We now show that this condition is met for the cases considered in this paper: considering a thickness of 10nm (typical for loosely packed chromatin) our polymer made of 500 beads describes a $L = 100 - 500$ kb segment (by coarse graining either 200 bp or 1 kbp into 10 nm). Typical TAD sizes are in the range 40 kb - 3 Mbp, with a median of 185 kb [23]. Thus our polymer represents a typical TAD in the scenario of loosest compaction (200 bp = 10 nm), or a large one in the case of tight compaction (1 kbp = 10 nm). In practice, we argue that in vivo chromatin compaction is heterogeneous, and that our polymer is well representative of a typical TAD in vivo.

With these numbers in mind, one should now consider typical extrusion (or diffusion) speeds of cohesin and condensin complexes in order to predict whether a SMC protein would be able to extrude a loop $\simeq L$ before disassociating at time τ. A useful parameter to bear in mind in this context is the processivity [14] $p = v\tau$ which captures the typical distance covered by a unidirectional SMC within time τ (for a diffusing SMC this parameter is equivalent to $p = \sqrt{D\tau}$).

SMC can actively or diffusively extrude loops comparable to large TADs before disassociating

We recall that recent experiments in vitro on condensin [33] measured an extrusion speed of at least 0.6 kb/s whereas indirect measurement using HiC in vivo obtained 0.2 kb/s [10] for eukaryotic and 0.9 kb/s [34] for bacterial condensin, respectively. It is thus ready to compute the range of distances travelled by condensin before disassociating (using $\tau = 20$ min): 240 kbp [10], 720 kbp [33] and 1Mbp [34]. These numbers are systematically larger than the size of chromatin fibre considered in this work which correspond to typical TADs in vivo. We thus argue that the assumption of permanently loaded SMC is a good approximation for typical TADs in vivo.

In the case of cohesin in interphase, in vitro experiments could not find unidirectional motion but measured an apparent diffusion constant of $1.72\mu m^2/s$ [35], $3.8\mu m^2/s$ [36] and $0.25\mu m^2/s$ [37]. A lower and upper bound of diffused lengths within $\tau = 20$ min and chromatin compaction $C = 0.1$ kb/nm (1kbp=10nm) are $p \simeq 1.7 - 6.7$ Mbp. [The minimum mobility to span 500 kbp in 20 minutes via diffusion would be $D = 0.02\mu m^2/s$ at this chromatin compaction]. Whereas for the loosest chromatin fibre $C = 0.02$ kb/nm (200 bp = 10 nm) the range of distance covered is $p \simeq 0.35 - 1.35$ Mbp.

As one can notice, diffusion of a cohesin over a chromatinised substrate can effectively span larger TADs than unidirectional condensin stepping (or hopping) on naked DNA [12]. In particular, we find that for both diffusing and actively extruding SMC, one can safely think SMC proteins to be permanently loaded over substrates with length of typical TADs in vivo (about 200 kbp). It is also intriguing to notice that "stripes" in HiC maps which are linked to cohesin are most abundant in TADs within this range of lengths [9].

This approximation breaks down at the length scale of very large TADs, as neither extruding nor diffusing cohesins can *systematically* cover length-scales of more than about $L \sim 1$ Mbp before disassociating. For this reason, in the next section we perform additional simulations of dynamically loaded SMC in order to study the limits of the proposed topological simplification mechanism and show that it is still valid in a stochastic sense rather than a systematic one.

Polymer Statistics and Unknotting via Processive SMC

In this section we discuss the results from several sets of simulations in which SMC proteins are dynamically loaded and unloaded at a certain rate $\kappa = 1/\tau$. Every time a SMC

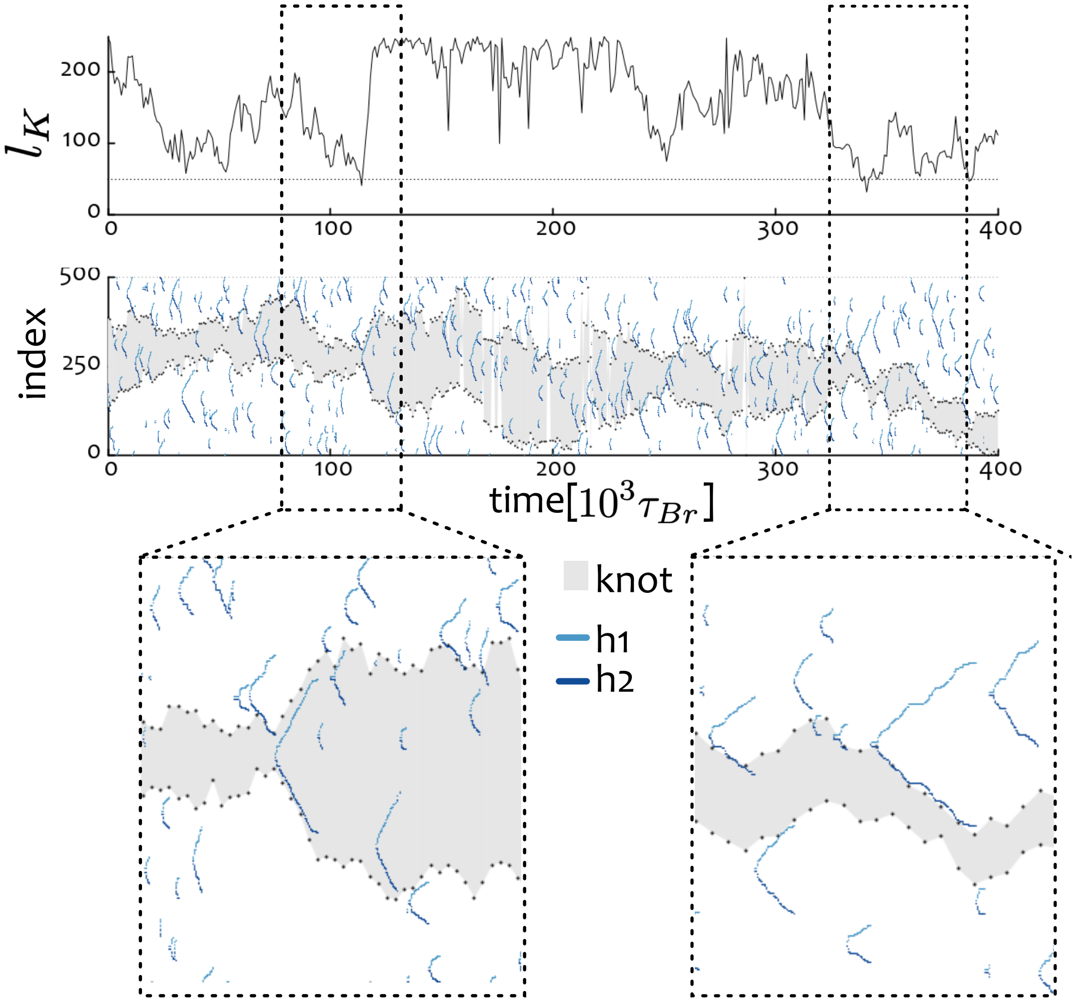

Figure S5: Randomly loaded SMC proteins with processivity shorter than the polymer size (here we set $p_{max} = 100\sigma$ and effective processivity $p \simeq 25\sigma$ on a polymer of length $L = 500\sigma$, see text for more details). The mid panel shows a kymograph of the knotted region (grey) and the position of SMC heads (blue). The top panel shows the instantaneous length of shortest knotted arc l_K (dashed line marks $l = 50$ beads). Bottom panels are zoom ins highlighting two different events: on the left a SMC is loaded within the minimum knotted arc and expands it, on the right several SMC are loaded on the flanks of the knot and reduce its size.

disassociates, we load a new one so that there is always one SMC bound at any one time (see Fig. S5 for an example of a kymograph). The disassociation time τ gives an upper bound on the length covered by an SMC with maximum speed v_{max}, which we here choose to be $v_{max} = 0.2\sigma/\tau_B$, i.e. an update on the position of both SMC heads is performed every 10 Brownian times. In this model, the *time* spent by any one SMC on the polymer follows a Poissonian statistics with mean τ, yet because of our conditional rule on the update move, the effective speed is $v < v_{max}$. This implies that the effective processivity is also shorter than the maximum one, i.e. $p = v\tau < p_{max} = v_{max}\tau$.

By measuring the distance covered by SMC at the unloading event, we recover the real distribution of lengths spanned alongside the real residency time of the SMCs. These are reported in Fig. S6 for two choices of τ. We observe that the effective speed $v \simeq 0.125 v_{max}$ so that the real SMC processivity p is about 8 times shorter than the one set externally, i.e. $p = p_{max}/8$.

As shown in Fig. S6, the statistics of unloading times and lengths correctly follows a Poissonian process; thus, the distribution of covered lengths is

$$P(l) = \frac{1}{p} e^{-l/p} \tag{10}$$

where p is the processivity. The probability to observe an event with residency time longer than τ' and hence length covered larger than l' is

$$p_{>l'} = \int_{l'}^{\infty} P(l)dl = 1 - \int_0^{l'} P(l)dl \tag{11}$$

and the typical number of events required to observe one such an event is simply $1/p_{>l'} = e^{l'/p}$.

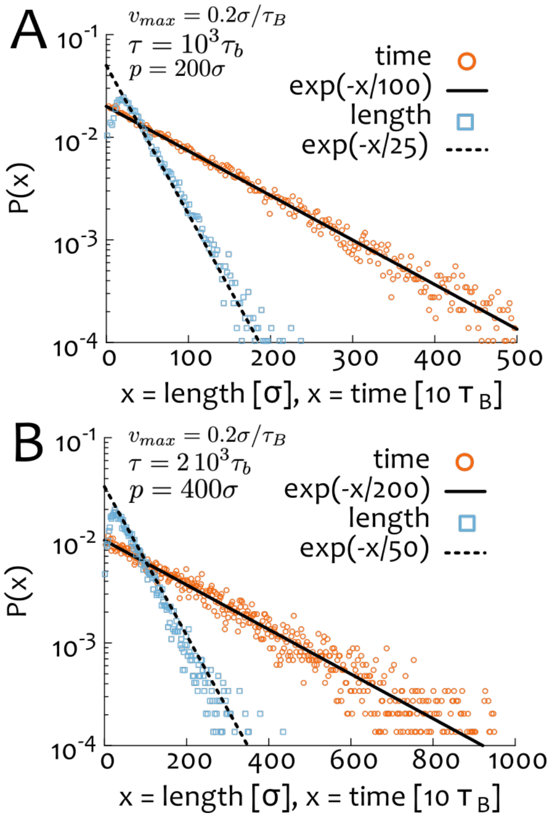

Figure S6: Distribution of SMC residency times (orange circles) follows an exponential decay with typical time (A) $\tau = 10^3\tau_B$ and (B) $\tau = 2\,10^3\tau_B$. The maximum speed is here set to $v_{max} = 0.2\sigma/\tau_B$ so that one expects a maximum processivity (A) $p = v\tau = 200\sigma$ and (B) $p = v\tau = 400\sigma$. The distribution of lengths covered by the SMC (blue squares) is instead captured by an exponential with effective processivity about (A) $p = 25\sigma$ and (B) $p = 50\sigma$ (corresponding to an effective velocity $v = 0.125 v_{max}$). The peak at short lengths is due to the chosen persistence lengths of about 20 beads.

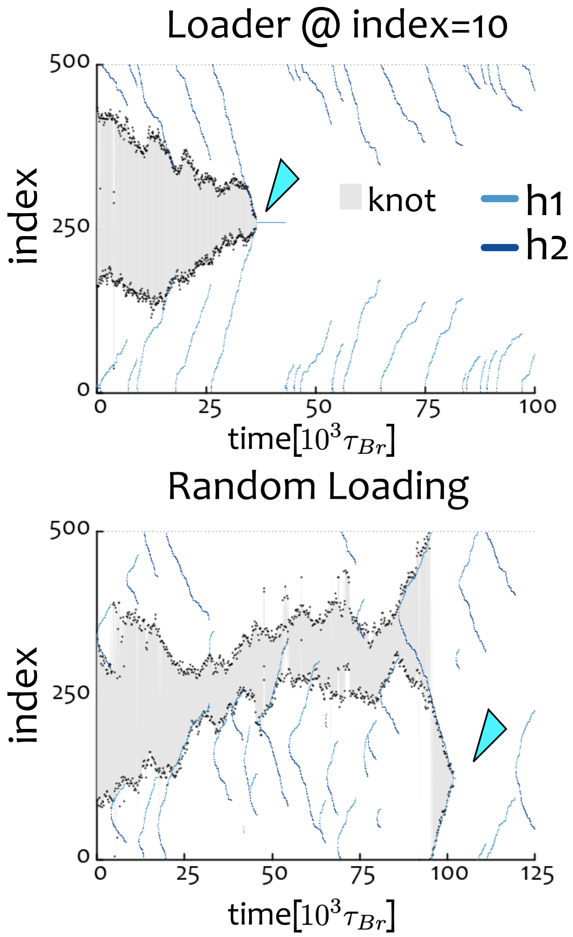

Figure S7: SMC proteins with effective processivity $p = 125\sigma < L = 500\sigma$ achieve stochastic efficient unknotting of a trefoil. The system needs to sample about $n_e = 1/p_{>L} = e^{L/p} \simeq 50$ events before observing one spanning the full contour. The figure shows a kymograph of the knotted region (grey) and the position of SMC heads (blue). The top panel shows the case of fixed loading (at bead 10) while the bottom panel shows the case with random loading. Arrowheads point to the unknotting event.

For instance, for $p = 30$ and $l' = 500$ one is required to sample $n_e \sim e^{17} \sim 25\,10^6$ events in order to observe one with processivity longer than 500 beads. For $p = 125$ this sampling number goes down to $n_e \simeq 50$, and indeed this is roughly what we find to be enough in order to untie a knotted polymer via SMC with effective processivity $p = 125 < L = 500$ (see Fig. S7).

Accordingly, to localise and then untie a knot on a 2 Mbp TAD through a SMC with speed 0.6 kbp/s and residency time $\tau = 20$ min one needs to sample on average 16 events, which may be roughly compatible with (if not underestimating) the number of cohesins loaded on a single TAD through interphase. We thus argue that while the approximation of infinite residency time is not kinetically accurate when $p < L$, the unknotting mechanism is still valid at times large enough to sample $e^{L/p}$ SMC loading/unloading events.

It is finally worth stressing that loading multiple (non-nested) SMC will linearly accelerate this process further.

Randomly-Bound versus SMC-Localised TopoII

To study the case in which TopoII is randomly bound on the substrate (Fig. 4 of main text) we performed at least 100 simulations for each value of TopoII density $\phi = 1, 10, 100\%$ and compared the knotting probability obtained by averaging over from 100 simulations done with a SMC-localised TopoII.

To do these simulations we started with a confined trefoil knot with $N = 500$ beads and either (i) loaded one extruding SMC at random and placed beads with soft interactions in front of SMC or (ii) loaded one extruding SMC at random and placed ϕN TopoII beads which we set as having soft interactions with the others.

As done in previous cases the SMC heads were updated every $10\,\tau_B$ steps, meaning a maximum speed $v_{max} = 0.2\sigma/\tau_B$ or a real speed of about $v = v_{max}/8$ (see above). In the case of dynamical and randomly bound TopoII, the soft-interacting

beads were set to be dynamically replaced along the substrate on average every $\tau_T = 10^3\tau_B$ meaning that the SMC complex can effectively cover a length $v_{max}\tau_T/8 \simeq 20\sigma$. For the case with $\phi = 1$ we simply set all the beads to be softly repelling all others, thus allowing bead-bead overlaps.

VIII. DIFFUSIVE SMCS CAN UNDO KNOTS BY LOCALISING ESSENTIAL CROSSINGS

To model diffusing SMCs, we update the position of the two heads independently, and move them either forward or backward with probability $1/2$. As before, the actual update is still conditional to the new Euclidean distance being shorter than the FENE bond. In order to speed up the simulations, we consider a shorter substrate ($L = 100$ beads $= 100$ kb) and perform update moves every Brownian time. This is equivalent to a maximum mobility of $1\sigma^2/\tau_{Br} = 200kbp^2/s = 0.02\mu m^2/s$ (using $\tau_{Br} = 0.5ms$ for a 10nm fibre), which is still smaller than that of cohesin in vitro [37]. We further recall that due to our conditional updating rule on the position of SMC heads, the actual simulated diffusion is even slower than this value.

We perform simulations starting from a trefoil knot, randomly load $N_L = 5$ SMC proteins and place 2 soft repulsive beads either (i) located in front of one of the N_L SMC (picked at random at every update timestep) or (ii) located randomly along the contour. In practice, to avoid numerical instabilities, case (ii) is modelled by setting a random pair of consecutive beads as "Topo2-active", i.e. subject to soft repulsive potential with the other beads with maximum energy barrier $\epsilon = 4k_BT$ and before returning them to the Lennard-Jones potential, they are transiently set to a "Topo2-removing" state in which they still interact via a soft potential but with a larger repulsive barrier ($\epsilon = 20k_BT$).

We discover that both sets of simulations yield to unknotting (see Fig. S8) and that this process is not anticipated by the localisation of the knotted arc, but by through the localisation of the essential crossings as also seen for the case of extruding SMCs (Fig. S3C-E). In Fig. S8C we show two consecutive snapshots in which the trefoil is being untied from a substrate with $N_L = 2$ diffusive SMCs.

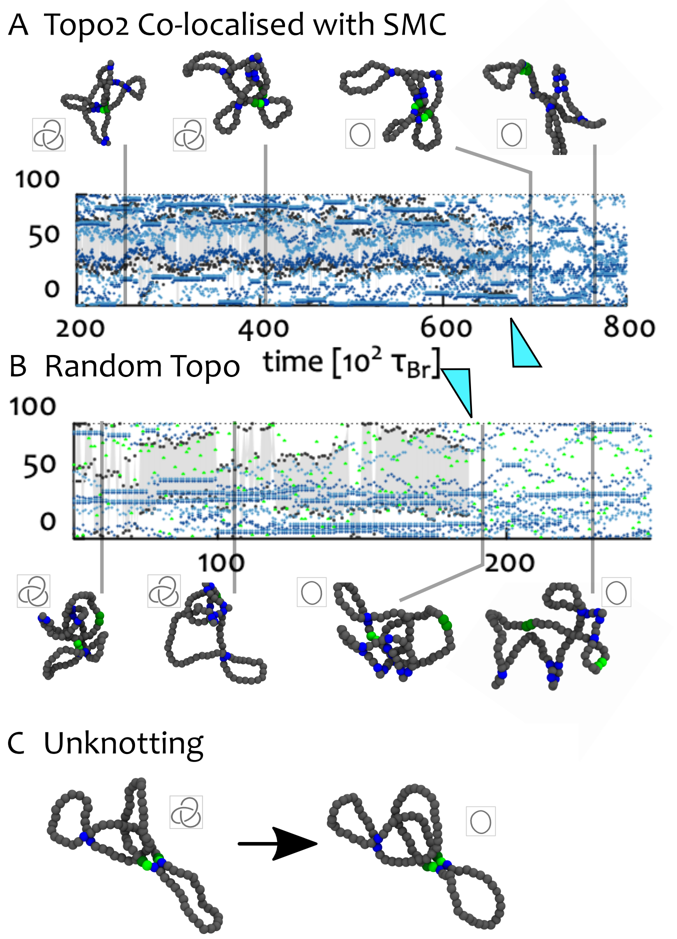

Figure S8: We perform simulations of diffusive SMC proteins on a 100 beads long polymer (tied in a trefoil knot) with co-localised (**A**) and random (**B**) action of Topo2. Both sets of simulations can yield unknotted substrates. Since we do not observe the localisation of the knotted arc, we argue that this unknotting pathway proceeds through the localisation of essential crossings, as more clearly illustrated in Fig. S3E. Thus, we require $N_L > 1$ in order to unknot polymers. In **C** we show an unknotting event with $N_L = 2$. In the snapshots, blue beads mark SMC heads while green beads are TopoII-bound segments.

IX. TABLE OF TRANSITIONS IN KNOT SPACE

In Table II we report a transition rates for all the topologies studied in this work. We recall that these transitions are calculated by performing at least 50 simulations initialised with a given topology. Every time the knot changes topology we record the event and finally compute the probability to end up in another knotted state. From the table it is evident that torus knots follow "cascades" whereas twist knots (7_2 and 5_2) have a non negligible probability to be unknotted in one step. Rates from random passage and hooked juxtaposition models are obtained from other works as shown in the table. These models extract transition rates on freely diffusing and flexible polymers. To faithfully compare transition rates in the confined case we thus performed simulations of the random passage model under the same confinement conditions as the ones for the synergistic case. See main text for detailed discussion.

X. MOVIES CAPTIONS

Colour scheme: Dark-grey beads mark the polymer backbone. Light-grey beads the beads belonging to the extruded portion of the polymer. Blue beads the location of SMC heads. Green beads the location of TopoII.

1. Supplementary Movie M1: Localisation of a trefoil

	Synergistic (this work)		RP		HJ	RP
	Free	Confined	Free Ref. [38]	Free Ref. [39]	Free Ref. [40]	Confined (this work)
$7_2 \to K$	0	0.02	–	0.5	–	–
$7_2 \to 5_2$	0.31	0.43	–	0.25	–	–
$7_2 \to 5_1$	0	0.02	–	0.005	–	–
$7_2 \to 0_1$	0.69	0.52	–	0.24	–	–
$7_1 \to K$	0.02	0.06	–	0.66	–	0.98
$7_1 \to 5_1$	0.98	0.92	–	0.34	–	0.02
$7_1 \to 3_1$	0	0.02	–	0	–	0
$6_1 \to K$	0	0.04	0.15	0.3	–	–
$6_1 \to 4_1$	0.39	0.33	0.53	0.44	–	–
$6_1 \to 0_1$	0.61	0.63	0.32	0.26	–	–
$5_2 \to K$	0	0.1	0.11	0.49	0.26	0.80
$5_2 \to 3_1$	0.5	0.25	0.53	0.2	0.23	0.13
$5_2 \to 0_1$	0.5	0.65	0.36	0.31	0.51	0.07
$5_1 \to K$	0	0.06	0.15	0.69	–	0.8
$5_1 \to 3_1$	1	0.94	0.85	0.31	–	0.13
$4_1 \to K$	0	0.04	0.08	0.16	–	0.84
$4_1 \to 0_1$	1	0.96	0.92	0.84	–	0.16
$3_1 \to K$	0	0.15	0.1	0.22	0.2	0.87
$3_1 \to 0_1$	1	0.85	0.9	0.78	0.8	0.13

Table II: Knot transition probabilities in different models. Topology simplification through the synergistic model proposed in this work is compared with RP (Ref. [39]) and HJ (Ref. [40]) models. The confined case is compared with RP simulations performed in this work. $\mathcal{K}_1 \to K$ denotes transition to any knot K with equal or larger minimal crossing number. (HJ, hooked juxtaposition; RP, Random Passage).

knot driven by a single processive SMC protein;

2. Supplementary Movie M2: Localisation of a Hopf link driven by a single processive SMC protein;

3. Supplementary Movie M3: Localisation and simplification of a trefoil knot via synergistic SMC-driven extrusion and Topo2-mediated strand crossing;

4. Supplementary Movie M4: Localisation of a trefoil knot under spherical confinement driven by a single processive SMC protein;

5. Supplementary Movie M5: Step-wise simplification of a 7_1 knot under spherical confinement;

6. Supplementary Movie M6: Simultaneous extrusion of two parallel SMC proteins localise essential crossings but stabilise a delocalised knotted state (shortest knotted arc is shown in cyan); this state can be simplified by TopoII recruited by SMC.

7. Supplementary Movie M7: Unknotting of a trefoil by two diffusing SMC proteins (blue beads). At each time step one SMC is selected at random, and a TopoII (green beads) is located in front of its motion.

[1] Rosa A, Everaers R (2008) Structure and dynamics of interphase chromosomes. *PLoS Comp. Biol.* 4(8):1.

[2] Sanborn AL, et al. (2015) Chromatin extrusion explains key features of loop and domain formation in wild-type and engineered genomes. *Proc. Natl. Acad. Sci.* 112(47):201518552.

[3] Goloborodko A, Marko JF, Mirny L (2015) Mitotic chromosome compaction via active loop extrusion. *bioRxiv* p. 021642.

[4] Michieletto D, Orlandini E, Marenduzzo D (2016) Polymer Model with Epigenetic Recolouring Reveals a Pathway for the de novo Establishment and 3D Organisation of Chromatin Domains. *Phys. Rev. X* 6:041047.

[5] Kremer K, Grest GS (1990) Dynamics of entangled linear polymer melts: A molecular-dynamics simulation. *J. Chem. Phys.* 92(8):5057.

[6] Calladine CR, Drew H, Luisi FB, Travers AA, Bash E (1997) *Understanding DNA: the molecule and how it works*. (Elsevier Academic Press) Vol. 1.

[7] Dekker J, Rippe K, Dekker M, Kleckner N (2002) Capturing chromosome conformation. *Science* 295(5558):1306–1311.

[8] Hirano T (2016) Review Condensin-Based Chromosome Organization from Bacteria to Vertebrates. *Cell* 164(5):847–857.

[9] Vian L, et al. (2018) The Energetics and Physiological Impact of Cohesin Extrusion. *Cell* 173:1–14.

[10] Gibcus JH, et al. (2018) A pathway for mitotic chromosome formation. *Science* 359(6376).

[11] Michieletto D (2016) On the tree-like structure of rings in dense solutions. *Soft Matter* 12:9485–9500.

[12] Brackley C, et al. (2017) Nonequilibrium Chromosome Looping via Molecular Slip Links. *Phys. Rev. Lett.* 119(13):138101.

[13] Uhlmann F (2016) SMC complexes: from DNA to chromosomes. *Nat. Rev. Mol. Cell. Biol.* 17(April).

[14] Fudenberg G, et al. (2016) Formation of Chromosomal Domains by Loop Extrusion. *Cell Rep.* 15(9):2038–2049.

[15] Rao SSP, et al. (2014) A 3D map of the human genome at kilobase resolution reveals principles of chromatin looping. *Cell* 159(7):1665–1680.

[16] Goloborodko A, Imakaev MV, Marko JF, Mirny LA (2016) Compaction and segregation of sister chromatids via active loop extrusion, Technical report.

[17] Baum M, Erdel F, Wachsmuth M, Rippe K (2014) Retrieving the intracellular topology from multi-scale protein mobility mapping in living cells. *Nat. Commun.* 5:4494.

[18] Michieletto D, Marenduzzo D, Orlandini E (2015) Is the kinetoplast DNA a percolating network of linked rings at its critical point? *Phys. Biol.* 12(1):036001.

[19] Plimpton S (1995) Fast Parallel Algorithms for Short-Range Molecular Dynamics. *J. Comp. Phys.* 117(1):1–19.

[20] Chen J, Englund PT, Cozzarelli NR (1995) Changes in network topology during the replication of kinetoplast DNA. *EMBO J.* 14(24):6339–6347.

[21] Alberts B, Johnson A, Lewis J, Morgan D, Raff M (2014) *Molecular Biology of the Cell.* (Taylor & Francis), p. 1464.

[22] Nora EP, et al. (2017) Targeted Degradation of CTCF Decouples Local Insulation of Chromosome Domains from Genomic Compartmentalization. *Cell* 169(5):930–944.e22.

[23] Rao SS, et al. (2017) Cohesin Loss Eliminates All Loop Domains. *Cell* 171(2):305–320.e24.

[24] Zirkel A, et al. (2017) Topological Demarcation By HMGB2 Is Disrupted Early Upon Senescence Entry Across Cell Types And Induces CTCF Clustering. *bioRxiv* 144.

[25] Tubiana L, Orlandini E, Micheletti C (2011) Multiscale entanglement in ring polymers under spherical confinement. *Phys. Rev. Lett.* 107(18):1–4.

[26] Suma A, Micheletti C (2017) Pore translocation of knotted DNA rings. *Proceedings of the National Academy of Sciences of the United States of America* 114(15):E2991–E2997.

[27] Gennes PGD (1979) *Scaling concepts in polymer physics*.

[28] Vologodskii A (2016) Disentangling DNA molecules. *Phys. Life Rev.* 18:118–134.

[29] Valdés A, Segura J, Dyson S, Martínez-García B, Roca J (2018) DNA knots occur in intracellular chromatin. *Nucleic Acids Res.* 46(2):650–660.

[30] Tedeschi A, et al. (2013) Wapl is an essential regulator of chromatin structure and chromosome segregation. *Nature* 501(7468):564–8.

[31] Hansen AS, Pustova I, Cattoglio C, Tjian R, Darzacq X (2017) CTCF and cohesin regulate chromatin loop stability with distinct dynamics. *Elife* 6:1–33.

[32] Busslinger GA, et al. (2017) Cohesin is positioned in mammalian genomes by transcription, CTCF and Wapl. *Nature* 544(7651):503–507.

[33] Ganji AM, et al. (2018) Real-time imaging of DNA loop extrusion by condensin. *Science* 7831(February):1–9.

[34] Wang X, Moazed D (2017) DNA sequence-dependent epigenetic inheritance of gene silencing and histone H3K9 methylation. *Science* 91(April):eaaj2114.

[35] Davidson IF, et al. (2016) Rapid movement and transcriptional re-localization of human cohesin on DNA. *EMBO J.* 35(24):2671–2685.

[36] Stigler J, Çamdere GÖ, Koshland DE, Greene EC (2016) Single-Molecule Imaging Reveals a Collapsed Conformational State for DNA-Bound Cohesin. *Cell Rep.* 15(5):988–998.

[37] Kanke M, Tahara E, Huis PJ, Nishiyama T (2016) Cohesin acetylation and Wapl-Pds 5 oppositely regulate translocation of cohesin along DNA. *EMBO J.* pp. 1–13.

[38] Flammini A, Maritan A, Stasiak A (2004) Simulations of action of DNA topoisomerases to investigate boundaries and shapes of spaces of knots. *Biophys. J.* 87(5):2968–75.

[39] Hua X, Nguyen D, Raghavan B, Arsuaga J, Vazquez M (2007) Random state transitions of knots: a first step towards modeling unknotting by type ii topoisomerases. *Topology and its Applications* 154(7):1381 – 1397. Special Issue: The Third Joint Meeting Japan-Mexico in Topology and its Applications.

[40] Burnier Y, Weber C, Flammini A, Stasiak A (2007) Local selection rules that can determine specific pathways of DNA unknotting by type II DNA topoisomerases. *Nucleic Acids Res.* 35(15):5223–5231.
